# Case Report: Clinical Features of Congenital Portosystemic Shunts in the Neonatal Period

**DOI:** 10.3389/fped.2021.778791

**Published:** 2021-12-02

**Authors:** Suhua Xu, Peng Zhang, Liyuan Hu, Wenhao Zhou, Guoqiang Cheng

**Affiliations:** Department of Neonatology, National Children's Medical Center, Children's Hospital of Fudan University, Shanghai, China

**Keywords:** congenital portosystemic shunts, abernethy syndrome, vascular malformations, neonate, case report

## Abstract

**Objective:** The aim of this single-center retrospective study was to analyze the clinical characteristics, treatment options, and course of neonatal-onset congenital portosystemic shunts (CPSS).

**Methods:** We included all patients with CPSS who presented with clinical symptoms within the neonatal period in our institution between 2015 and 2020.

**Results:** Sixteen patients were identified, including 13 patients with intrahepatic portosystemic shunts (IPSS) and three patients with extrahepatic portosystemic shunts (EPSS). The median age of diagnosis was 16 days (range prenatal 24 weeks−12 months). Hyperammonemia (60%), neonatal cholestasis (44%), elevated liver enzyme (40%), hypoglycemia (40%), thrombocytopenia (38%), and coagulation abnormalities (23%) appeared in neonatal CPSS. Twelve patients (75%) presented with congenital anomalies, of which congenital heart disease (CHD) (44%) was the most common. Thirteen patients with IPSS initially underwent conservative treatment, but two of them were recommended for the catheter interventional therapy and liver transplantation, respectively, due to progressive deterioration of liver function. Spontaneous closure occurred in nine patients with IPSS. The shunt was closed using transcatheter embolization in one patient with EPSS type II. Another patient with EPSS type II underwent surgical treatment of CHD firstly. The remaining patient with EPSS type Ib received medical therapy and refused liver transplantation.

**Conclusion:** Hyperammonemia, neonatal cholestasis, elevated liver enzyme, hypoglycemia, and thrombocytopenia are the main complications of neonatal CPSS. Moreover, CPSS is associated with multiple congenital abnormalities, especially CHD. Intrahepatic portosystemic shunts may close spontaneously, and conservative treatment can be taken first. Extrahepatic portosystemic shunts should be closed to prevent complications.

## Introduction

Congenital portosystemic shunt (CPSS) is a rare vascular malformation in which the venous blood from the intestines and spleen bypasses the liver and diverts directly into the systemic circulation through abnormal vessels. Although the overall incidence is unknown, some studies estimated that about 1/30,000 newborns are affected through the hereditary galactosemia screening test (1). With the improvement of imaging techniques and further recognition of CPSS, the number of reported cases has been increasing since first reported by Abernethy in 1793 (1–3).

Congenital portosystemic shunt has been associated not only with congenital malformations, like congenital heart disease (CHD), and Down syndrome, but also with multisystem complications including antenatal hemodynamic abnormalities, neonatal cholestasis, hepatopulmonary syndrome, pulmonary hypertension, and even hepatic tumors. The appropriate treatment strategies of CPSS, including conservative therapy, transcatheter embolization, shunt vessel ligation, and liver transplantation, should take into account many factors, mainly the shunt type, shunt ratio, patient age, and the presence of complication (1–3). The intrahepatic portosystemic shunts (IPSS) sometimes can close spontaneously before the age of 2 years, but others, especially extrahepatic portosystemic shunts (EPSS), persist throughout life and carry risks of severe life-threatening complications, especially after the third year of life (1, 4). Therefore, early detection, systematic evaluation, and appropriate intervention are essential for a good prognosis. This study aimed to retrospectively analyze the clinical features, diagnosis, therapeutic treatment, and outcomes of the patients with CPSS who developed symptoms from the neonatal period.

## Methods

This was a single-center retrospective study. We reviewed the electronic medical records of the Children's Hospital of Fudan University between 2015 and 2020 to identify all consecutive patients with CPSS.

The inclusion criteria in our study were patients aged 28 days or less at the time of the first symptoms. We collected the following data through the electronic medical record: history of pregnancy and delivery, gender, gestational age at birth, birth weight, age at diagnosis of CPSS, clinical manifestation, laboratory and imaging results, genetic test results, clinical interventions, and outcomes.

**Figure 1:**
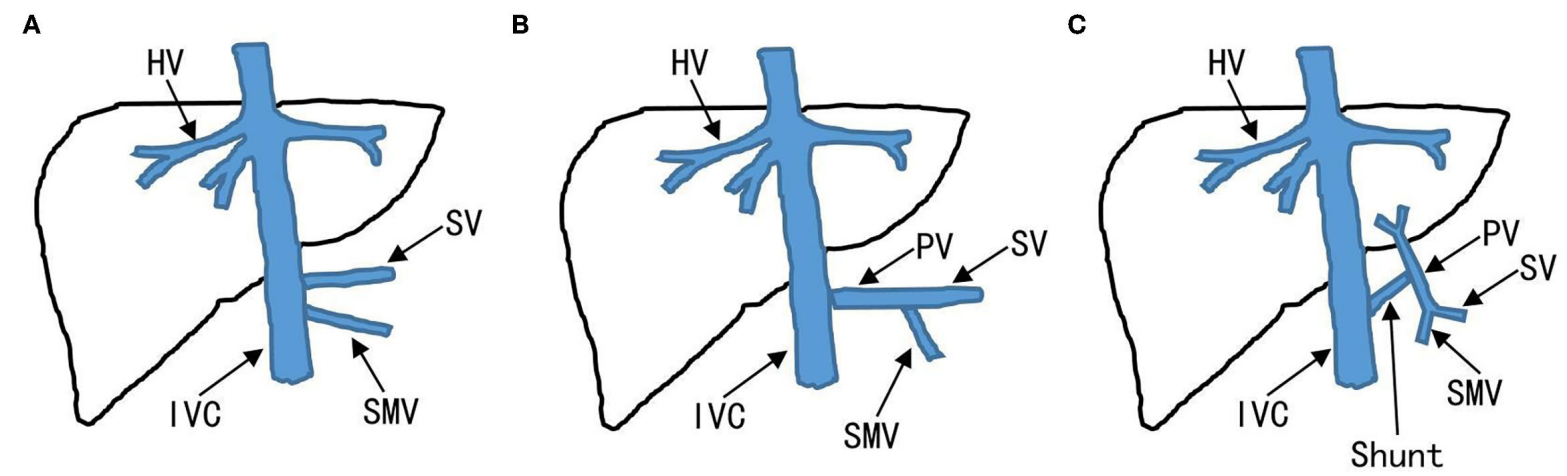
Classification of EPSS. Absence of the PV to the liver is defined as EPSS type I. **(A)** EPSS type Ia: the SV and SMV drain respectively into IVC. **(B)** EPSS type Ib: the SV and SMV confluent into the IVC. **(C)** EPSS type II: existence of the PV to the liver. EPSS, extrahepatic portosystemic shunts; IVC, inferior vena cava; PV, portal veins; SMV, superior mesenteric vein; SV, splenic vein.

The CPSS was classified as IPSS and EPSS based on abdominal ultrasonography (US), contrast-enhanced computerized tomography (CT), contrast-enhanced magnetic resonance imaging (MRI), digital subtraction angiography (DSA), or a combination of these techniques. Extrahepatic portosystemic shunts also called “Abernethy malformations” is further classified into types I and II (Figure 1). Intrahepatic portosystemic shunts is not limited to one lobe of the liver and can be composed of multiple portosystemic connections.

Quantitative variables were expressed as median and range, while qualitative variables were expressed as a percentage.

The study was conducted in accordance with the Helsinki Declaration and approved by the Ethics Committee of the Children's Hospital of Fudan University.

## Results

Sixteen consecutive patients were identified, including two preterm infants (13%, 2/16) and 13 males (81%, 13/16) (Table 1). The median age of diagnosis was 16 days (range from prenatal 24 weeks to 12 months). Eleven patients were diagnosed at an age younger than 1 month, including three diagnosed prenatally (Table 2). The IPSS was found in 13 patients (including three cases of patent ductus venosus), 85% (11/13) of whom were male. Three patients had an extrahepatic shunt including one type Ib and two type II (Table 2).

**Table 1:** The clinical and laboratory features of 16 patients with CPSS in the neonatal period.

**Case**	**Sex**	**GA (week)**	**Clue for** **investigation**	**Fetal** **US findings**	**TB/DB** **(μmol/L)**	**ALT (IU/L)**	**GGT (IU/L)**	**TBA (μmol/L)**	**Ammonia (μmol/L)**	**PT (s)**	**Blood glucose (mmol/L)**	**PLT (×10^**9**^/L)**	**Other clinical presentation**	**Associated anomalies**
1	M	38	Jaundice	Normal	249/17	8.1	303.5	17.6	NA	14	NA	66	None	Down syndrome, ASD, PDA
2	M	37	Abnormity of prenatal US	UV-HPV shunt; IUGR	410/9	10.1	106.2	67.2	NA	14	5.16	176	None	None
3	M	39	Jaundice	Normal	408/29	12	231	64.8	125.6	15	4.6	253	None	Hypospadias
4	M	38	Jaundice	Normal	267/11	12	60	6.8	114	16	0.9	185	Neonatal seizure	Splenulus
5	M	37	Abnormity of prenatal US	Hepatomegaly, hydropericardium, polyhydramnios, intrahepatic UV varix	265/20	19.5	322.8	17	86	13	4.5	170	None	Noonan syndrome-8, pulmonary stenosis, ASD, PDA, hypertrophic cardiomyopathy
6	F	39	Thrombocytopenia	IUGR	161/12	6	56	42.2	NA	27	2.1	41	None	PDA
7	M	35	Abnormity of prenatal US	Intrahepatic shunt	115/62	71	50	47.4	83	15	4.9	267	None	None
8	M	37	Jaundice	IUGR	127/72	125.2	110.3	136.7	92	15	4.4	258	Transient acholic stool	None
9	M	37	Abnormity of prenatal US	Intrahepatic shunt; IUGR	165/93	165	126	125.9	159	16	3.2	252	None	ASD, metabolic osteopathy
10	F	38	Jaundice	Normal	414/293	205	38.4	77.8	166	16	0.8	50	None	None
11	M	38	Jaundice	Normal	697/436	223	NA	NA	192.5	>100	NA	20	AUGIB, respiratory failure	Hypertrophic cardiomyopathy
12	M	40	Jaundice	Normal	290/239	272.9	37	104.2	51	14	NA	218	Neonatal seizure	Intracranial vascular malformation
13	M	34	Jaundice	Oligohydramnios	310/109	18.9	52.2	245.1	336	>100	2	60	Respiratory failure, AUGIB	PDA
14	M	39	Jaundice	Normal	278/36	17	NA	NA	NA	NA	NA	NA	None	Right cryptorchism, right oblique inguinal hernia
15	M	40	Multiple congenital malformations	Oligohydramnios	256/31	NA	NA	NA	NA	NA	NA	NA	None	Adams-Oliver syndrome 5, VSD, PDA, partial absence of right parietal bone, splenulus
16	F	39	Jaundice	IUGR	213/24	11	NA	NA	NA	NA	NA	NA	None	Aproctia, scoliosis, butterfly vertebra, right ventral hernia

*ALT, alanine aminotransferase; ASD, atrial septal defect; AUGIB, acute upper gastrointestinal bleeding; CPSS, congenital portosystemic shunts; DB, direct bilirubin; F, female; M, male; NA, not available; GA, gestational age; GGT, glutamyltranspeptidase; HPV, hepatic portal vein; IUGR, intrauterine growth restriction; PDA, patent ductus arteriosus; PLT, platelet count; PT, prothrombin time; TB, total bilirubin; US, ultrasonography; UV, umbilical vein; VSD, ventricle septal defect*.

**Table 2:** The shunt characteristics, treatment, and outcome of 16 patients with CPSS.

**Case**	**Age at diagnosis**	**Type of shunt**	**Anatomy**	**Imaging methods**	**Characteristics after** **neonatal period**	**Treatment**	**Outcome**
1	19 days	IPSS	LPV-IVC	US	None	Conservative	Age 8 months: shunt present on US; normal liver tests
2	29 weeks (GA)	IPSS	LPV-MHV	US/CT	None	Conservative	Age 10 months: disappearance of shunt on CT; normal liver tests
3	13 days	IPSS (PDV)	LPV-LHV	US/CT	None	Conservative	Age 13 months: disappearance of shunt on US; normal liver tests
4	7 days	IPSS	LPV-MHV	US/CT	None	Conservative	Age 36 months: disappearance of shunt on US; normal liver tests
5	7 days	IPSS	LPV-IVC	US/CT	None	Conservative	Age 12 months: shunt present on US; normal liver tests; failure to thrive
6	6 days	IPSS	LPV-RHV, multiple	US/CT	None	Conservative	Age 6 months: disappearance of shunt on US; normal liver tests
7	34 weeks (GA)	IPSS (PDV)	LPV-MHV	US/CT	None	Conservative	Age 13 months: disappearance of shunt on US; normal liver tests
8	9 days	IPSS (PDV)	LPV-MHV	US/CT	None	Conservative	Age 3 months: disappearance of shunt on US; normal liver tests
9	24 weeks (GA)	IPSS	LPV-LHV, MHV	US/CT	None	Conservative	Age 4 months: disappearance of shunt on US; normal liver tests
10	20 days	IPSS	LPV-LHV, multiple	US/CT	None	Conservative	Age 4 months: disappearance of shunt on CT; age 6 months: normal liver tests
11	3 months	IPSS	LPV-MHV	US/CT	Age 3 months: hepatic function improvement	Conservative	Age 12 months: disappearance of shunt on US; normal liver tests
12	1.1 months	IPSS	LPV-MHV, multiple	US/MRI	Age 48 days: deterioration of liver function	Conservative treatment first, the interventional treatment recommended	Loss of follow-up after automatic discharge at age 1.7 months
13	19 days	IPSS	LPV-LHV	US/CT	NA	Conservative treatment first, hepatic transplantation recommended	Loss of follow-up after automatic discharge at age 1 month
14	3.2 months	EPSS type II	SV-LRV	US/CT/DSA	Age 35 days: TB/DB 232/156.6 μmol/l, ALT 257 U/l; age 2.4 months: hepatosplenomegaly, hypoglycemic episodes	Embolization with balloon at age 10 months	Age 13 months: disappearance of shunt on CT; normal liver tests
15	12 months	EPSS type II	RPV-IVC	US/CT/DSA	Age 11 months: hepatosplenomegaly; age 12 months: TB/DB 23.9/10.6 μmol/l, pulmonary hypertension, cardiac insufficiency, failure to thrive	Conservative; cardiac surgery at age 12.5 months	Loss of follow-up after discharge at age 13 months
16	4.2 months	EPSS type Ib	PV–IVC	US/CT/DSA	Age 2 months: TB/DB 189.5/117.7 μmol/l, ALT 447 IU/l, GIB	Conservative treatment, hepatic transplantation recommended	At 56 months: growth retardation, hepatic encephalopathy, coagulation abnormalities

*ALT, alanine aminotransferase; CPSS, congenital portosystemic shunts; CT, computerized tomography; DB, direct bilirubin; Dx, diagnosis; DSA, digital subtraction angiography; EPSS, extrahepatic portosystemic shunts; GA, gestational age; GIB, gastrointestinal bleeding; HPV, hepatic portal vein; IPSS, intrahepatic portosystemic shunts; IVC, inferior vena cava; PDV, patent ductus venosus; PV, portal vein; LPV, left portal vein; LHV, left hepatic vein; LRV, left renal vein; MHV, middle hepatic vein; MRI, magnetic resonance imaging; NA, not available; RPV, right portal vein; RHV, right hepatic vein; SV, splenic vein; TB, total bilirubin; UV, umbilical vein; US, ultrasonography*.

### Clinical Manifestation in the Neonatal Period

Intrauterine growth restriction (IUGR) was found in five patients (31%, 5/16). Three patients (19%, 3/16) had prenatal hemodynamic abnormalities, two of whom had oligohydramnios. In the remaining one patient, prenatal US showed hepatomegaly, intrahepatic umbilical vein varix, hydropericardium, and polyhydramnios, and fetal chromosome karyotype analysis indicated no obvious abnormality.

All patients (100%, 16/16) presented with jaundice during the neonatal period, but only one had a transient acholic stool. Acute upper gastrointestinal bleeding (AUGIB) was discovered in two patients with respiratory failure. Neonatal seizure was noted in two patients, which disappeared after anticonvulsant therapy.

Twelve patients (75%, 12/16) presented with congenital anomalies, including CHD (44%, 7/16), abdominal malformations (31%, 5/16), genetic syndromes (19%, 3/16), orthopedic anomalies (19%, 3/16), urogenital malformations (13%, 2/16), and cerebrovascular malformation (6%, 1/16). One patient was identified during the evaluation of multiple malformations. For details, see Table 1.

### The Laboratory Results in the Neonatal Period

Hyperbilirubinemia developed in all patients during the neonatal period, and the median level of total bilirubin was 266 μmol/l (range from 115 to 697). The direct bilirubin (DB) levels were increased in 7 of 16 patients (median: 109 μmol/l, range 62–436). Alanine aminotransferase (ALT) levels were elevated in 6 of the 15 patients (median: 4.6 × upper limit of normal, range 1.8–6.8 × upper limit of normal), serum γ-glutamyltranspeptidase levels were elevated in 6 of 12 patients reported, and high total serum bile acid (TBA) concentration in 11 of the 12 patients. Prothrombin time (PT) was significantly prolonged in 3 of the 13 patients even with vitamin K administration. Six of the 10 patients had ammonia levels greater than 110 μmol/l. Forty percent (4/10) of patients experienced severe hypoglycemia (<2.2 mmol/l), and all except one (Case 13) gradually returned to normal blood-glucose levels after increasing the intravenous infusion rate and establishing enteral feeding. Thirty-eight percent (5/13) of patients had thrombocytopenia (range 20–66 × 10^9^/l).

### Diagnosis

In all but one patient, CPSS was screened by US and then confirmed by contrast-enhanced CT or contrast-enhanced MRI or DSA. Significantly, two IPSSs (Case 10, 11) and one EPSS type II (Case 14) were missed by abdominal US and later detected by contrast-enhanced CT or MRI. Reasons for the missed diagnosis were mentioned in the section Discussion. In addition, one patient (Case 15) was misdiagnosed as having portal vein (PV) absence by US and contrast-enhanced CT, but the main right PV was developed in DAS. For details, see Table 2.

### Treatment and Outcome

Conservative treatment was initially performed in 13 patients with IPSS. Hyperbilirubinemia, abnormal ALT, prolonged PT, elevated ammonia, hypoglycemia, and thrombocytopenia resolved spontaneously in 11 patients at a median age of 1.2 months (range 0.3–5). Spontaneous closure occurred in nine patients with intrahepatic shunts at a median age of 10 months (range 3–36). The abdominal US showed the persistence of shunt in two patients at ages 8 and 12 months, but liver function tests are normal so far.

Conservative treatment failed in two patients with IPSS (Case 12 and 13). Case 12 underwent progressive deterioration of liver function (ALT 561 IU/l, DB 202.5 μmol/l, blood ammonia 133 μmol/l, TBA 108.2 μmol/l) on day 48 after birth. Case 13 developed enzyme bilirubin separate, intractable coagulation disorder, and hyperammonemia during the neonatal period secondary to CPSS. The catheter-based intervention and liver transplantation were recommended, respectively, but the two patients left the hospital voluntarily and lost to follow-up.

Three patients with EPSS (Cases 14–16) presented with hyperbilirubinemia (predominantly in DB) in the neonatal period, followed by cholestasis and abnormal liver function. All other causes of cholestasis and liver dysfunction were excluded. Case 14 developed hepatosplenomegaly and hypoglycemic episodes at the age of 2.4 months. He underwent a percutaneous embolization with an umbrella device of his shunt at age of 10 months, and his abnormal blood chemical features returned to normality 3 months after successful shunt closure. Case 15 was found to have hepatosplenomegaly at an age of 11 months. He developed pulmonary hypertension and cardiac insufficiency (NYHA classes II–III) at diagnosis of CPSS (12 months of age). Cardiac catheterizations showed that the mean pulmonary artery pressure was 47 mmHg. So he underwent surgical treatment of CHD (tricuspid valvuloplasty, ligation of patent ductus arteriosus, repair of ventricle septal defect, and patent foramen ovale) rather than shunt closure at our hospital and was lost to follow-up. Case 16 with PV absence developed gastrointestinal bleeding at 2 months of age and growth retardation, hepatic encephalopathy, and coagulation abnormalities at 56 months of age (last follow-up visit) who refused liver transplantation and was treated with medical therapy. For details, see Table 2.

## Discussion

We reported 16 patients with CPSS who presented with symptoms during the neonatal period, including 13 IPSSs. As far as we know, this is the first series report about the clinical features of neonatal-onset CPSS. Our results show that not only hyperammonemia, neonatal cholestasis, and elevated liver enzyme but also hypoglycemia and thrombocytopenia are the main complications. Our series confirm that CPSS is associated with congenital malformations and that most of IPSSs have a good prognosis.

With the development of prenatal imaging, the number of CPSS diagnosed prenatally is increasing (3, 5). In our study, three patients were prenatally diagnosed through US. Francois et al. (3) show that abnormal prenatal hemodynamics (pericardial effusion, dilated cardiomyopathy, and tricuspid insufficiency) is the most frequent complication (41%, 27/66) associated with CPSS. In addition, an abnormal intrahepatic duct or anechoic tubular, a dilated hepatic vein (HV), atypia of liver size, other prenatal hemodynamic abnormalities (e.g., oligohydramnios, polyhydramnios, elevated abdominal circumference during the third trimester), and CHD detected by prenatal US are important clues for identifying CPSS (3, 5, 6). In our study, two patients had oligohydramnios, and one patient presented with hepatomegaly, intrahepatic umbilical vein varix, hydropericardium, and polyhydramnios on prenatal US. Francois et al. (3) found that children with CPSS diagnosed prenatally appear to have fewer complications of the shunt, possibly due to the spontaneous and/or prophylactic shunt closure or early termination of pregnancy in more severe cases. In our study, all patients diagnosed prenatally with CPSS have good outcomes. Moreover, when CPSS is diagnosed during the fetal period, the algorithm for the perinatal management of CPSS proposed by Francois et al. (3) should be carried out to detect complications and other anomalies associated with CPSS.

Congenital portosystemic shunts is rare but associated with multiple congenital abnormalities such as cardiovascular, neurologic, digestive, and urinary systems (1–3). In our study, 75% (12/16) of patients had congenital anomalies, a rate in accordance with 65% (80/123) of patients with CPSS diagnosed prenatally (3). Congenital heart disease is the most common anomaly associated with CPSS (ranging from 17 to 30%) (1–3, 5, 7–9), which was discovered in 44% (7/16) patients in our study. In fact, these malformations, including CPSS, may be part of a syndrome or genetic abnormality or complex congenital malformative process such as Down syndrome, Trisomy 18, Turner syndrome, Noonan syndrome, and Bannayan–Zonana syndrome (1–3). Lambert et al. (5) found that left heterotaxy with complex cyanotic heart disease and polysplenia syndrome presented in 15% of patients with CPSS and at least one cardiovascular disorder, which is very rare in the general population (10). In view of this close association, screening for CPSS should be performed in neonates with CHD, especially those with multiple malformations.

Meanwhile, CPSS has a variety of clinical features resulting from different pathophysiologies. The presence of a shunt reduces PV blood flow. Intrauterine growth restriction is associated with CPSS (ranging from 17 to 50%) (1, 3, 11), which may be related to liver malnutrition due to PV ischemia. In our study, 31% (5/16) of patients had IUGR. Hepatic hypoperfusion has been known to increase the risk of neonatal cholestasis (12). Anoxic–ischemic neonatal cholestasis is quite characteristic for CPSS (1, 13). Forty-four percent (7/16) of patients had cholestasis during the neonatal period in our study. However, it is worth mentioning that the three patients diagnosed with EPSS mainly showed indirect hyperbilirubinemia in the neonatal period, and jaundice progressively worsened or recurred after conservative treatment and gradually developed cholestasis.

Congenital portosystemic shunts can also cause the diversion of metabolites directly into the systemic circulation without liver metabolism, resulting in clinical symptoms, and resultant laboratory alterations. Investigation of a positive screening test for galactosemia after birth is the main clue for the diagnosis of CPSS in the neonatal period in some countries (1, 2). However, we do not routinely screen for galactosemia which is rare in China (14). None of the patients in our study underwent this test in the neonatal period. Hyperammonemia is common among patients with CPSS (1–3, 8), and it was present in 60% (6/10) of newborns in our case series. Hypoglycemia seems to be a rare complication of CPSS, which is most likely due to hyperinsulinism from decreased hepatic insulin degradation (15). It has been reported mostly in newborns, in whom it can be severe and persistent (1, 8, 15). In our study, 40% (4/10) of patients experienced severe neonatal hypoglycemia, one of whom (Case 13) had recurrent episodes of hypoglycemia despite aggressive medical treatment. In addition, one patient (Case 14) in our study was accidentally found to have hypoglycemia episodes in infancy, although the level of blood glucose in the neonatal period was unknown. Our cases further provided evidence to support the proposals of Weigert et al. (15) that persistent hypoglycemia is one of the reasons for CPSS screening and persistent screening of hypoglycemia in CPSS patients.

The presence of a shunt can also impair liver synthesis (1, 16), which can lead to coagulopathy due to insufficient production of coagulation factors. Prothrombin time was prolonged in 31 of 77 children reviewed by Bernard et al. (1) and in 3 of 13 neonates from our cases. Neonatal thrombocytopenia, one of the most common hemorrhagic disorders in newborns, has a wide range of possible etiologies (17). Thrombocytopenia in children with CPSS has been reported in the previous literature (1, 3, 7), but the exact pathophysiology has not been ascertained. It has been suggested that hepatic thrombopoietin production is regulated by the portal blood flow into the liver (18). One may postulate that the thrombocytopenia in children with CPSS is related to the insufficient synthesis of thrombopoietin due to PV blood flow alteration. Thirty-eight percent (5/13) of the neonates presented with thrombocytopenia in our study. Intrahepatic shunt in Case 6 was discovered during the search for the etiologies of thrombocytopenia. We observed a continuous decrease of platelets and further deterioration of coagulation function in two neonates (Case 11 and 13) with worsening liver function, and both of the above two neonates had AUGIB during the course of the disease. Therefore, CPSS can be an important consideration in the differential diagnosis of neonatal thrombocytopenia and/or coagulopathy. Hepatopulmonary syndrome, portal pulmonary hypertension, and hepatic encephalopathy are the most prominent manifestations secondary to long-term portosystemic shunting and are not observed in patients during the neonatal period in our study.

Because of the advantages of convenient operation and non-invasive and non-ionizing radiation, US is the initial imaging modality for the diagnosis of CPSS (1, 19). However, it may not accurately demonstrate the associated intrahepatic and extrahepatic shunts due to limited imaging features, gastrointestinal gas interference, etc. Shunts between the PV branch and the HV or the ductus venosus can be easily diagnosed by US as dilation of the two communicating vessels (1). In our study, the shunts joining the left PV branch to the left PV in Case 10 were diagnosed as ectasia of the PV by US. As color Doppler US only assesses the PV and HV and their branches, extrahepatic shunts far from the liver, like the shunt between the splenic vein and left renal vein in Case 14, may be difficult to detect. Other US findings in CPSS can be non-specific, such as hepatomegaly and uneven echogenic liver parenchyma in Case 11. Contrast-enhanced CT and MRI can not only detect the anatomy and location of the shunt and other abdominal vessels but also observe the lesions of abdominal solid organs and other accompanying abnormalities. Therefore, contrast-enhanced CT or MRI are used to confirm the diagnosis at a later stage (1, 19), especially when clinicians suspect CPSS, but US result is negative or non-specific. Angiography helps to further calibrate the venous communication, especially when PV and its intrahepatic branches are not visible on contrast-enhanced CT and MRI due to the significant decrease of intrahepatic PV blood flow (1, 19). It can more accurately demonstrate the presence and location of PV and the degree of hypoplasia of the intrahepatic portal branches. As in Case 15 in our study, the main right branch of PV was shown on PV angiography but not on contrast-enhanced CT.

**Figure 2:**
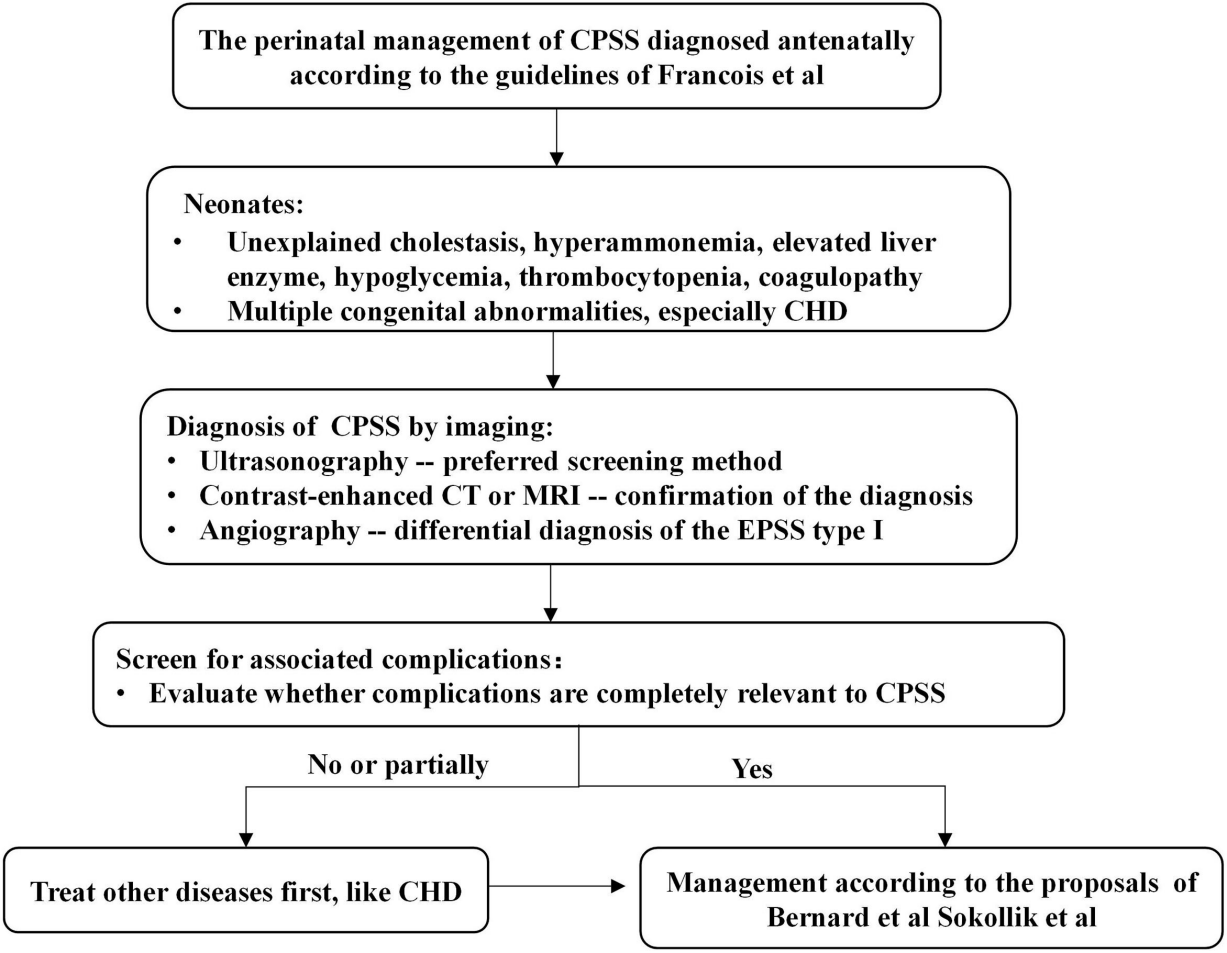
Algorithm for the management in a neonate with CPSS. Francois et al. (3), Bernard et al. (1), and Sokollik et al. (2). CHD, congenital heart disease; CPSS, congenital portosystemic shunts; CT, computerized tomography; MRI, magnetic resonance imaging.

According to the previous literature reviews (1–3, 13), the spontaneous closure rate of CPSS varies greatly between 5 and 52%, depending on the location and size of the shunt. Small IPSSs tend to disappear spontaneously during the first 2 years of life without major complications, while large IPSSs and EPSSs persist and carry risks of complications. Algorithms for managing CPSS later in infancy proposed by Bernard et al. (1) and Sokollik et al. (2) recommend that closure of the shunt should be scheduled earlier in case of severe complications or if the IPSSs have not disappeared at around 2 years of age. In our study, the majority of patients diagnosed with IPSS during the neonatal period closed spontaneously and had a good outcome on conservative treatment. However, when patients with CPSS develop severe complications or fail to close shunt spontaneously, we need to determine whether these clinical symptoms are entirely caused by CPSS or by other anomalies. Lambert et al. (5) found that heart failure was the main symptom in CPSS children with cardiovascular disorders (especially neonates with hemodynamically significant CHD) during the first months of life. However, heart failure symptoms of these neonates gradually improved under conservative treatment and/or after cardiac surgery. Additionally, Fugelseth et al. (20) and Poeppelman et al. (8) demonstrate that a prostaglandin E infusion and high right-sided pressure related to CHD or pulmonary hypertension may contribute to the patency of the ductus venosus or its clinical significance. Due to concern that complex CHD was one of the important factors leading to pulmonary hypertension and cardiac insufficiency, Case 15 was first treated with cardiac surgery. However, interventional or surgical closure of the shunt and even liver translation may be necessary if the severe clinical status is entirely attributable to CPSS and conservative treatment is ineffective, as in cases 12–14 in our series. Thus, based on previously reported literature and the results of our study, we propose a management algorithm for neonates with CPSS (Figure 2).

In summary, CPSS should be considered as one of the important differential diagnoses of unexplained hepatic dysfunction (neonatal cholestasis, elevated liver enzymes, hyperammonemia, or coagulopathy), hypoglycemia, and thrombocytopenia in neonates, especially when CHD or other congenital malformations are present. US is the initial screening method, followed by contrast-enhanced CT or contrast-enhanced MRI, or even DSA, to confirm the diagnosis. The majority of the neonatal IPSSs have good outcomes, and conservative treatment can be taken first. EPSS, however, should be considered for closure to prevent complications.

## Data Availability Statement

The original contributions presented in the study are included in the article/supplementary material, further inquiries can be directed to the corresponding author/s.

## Ethics Statement

The studies involving human participants were reviewed and approved by the Ethics Committee of Children's Hospital of Fudan University. Written informed consent from the participants' legal guardian/next of kin was not required to participate in this study in accordance with the national legislation and the institutional requirements.

## Author Contributions

Material preparation, data collection, and analysis were performed by SX, PZ, and LH. The first draft of the manuscript was written by SX. All authors contributed to the study conception and design, commented on previous versions of the manuscript, and read and approved the final manuscript.

## Funding

This research was supported by Big Data and Artificial Intelligence Research Funding Project of Clinical Research Center of National Children's Medical Center (No. 2020DSJ13).

## Conflict of Interest

The authors declare that the research was conducted in the absence of any commercial or financial relationships that could be construed as a potential conflict of interest.

## Publisher's Note

All claims expressed in this article are solely those of the authors and do not necessarily represent those of their affiliated organizations, or those of the publisher, the editors and the reviewers. Any product that may be evaluated in this article, or claim that may be made by its manufacturer, is not guaranteed or endorsed by the publisher.
